# A New Feasible Opportunity for Recycling Lead and Silver from Zinc Plant Residues by Flotation

**DOI:** 10.3390/ma17215218

**Published:** 2024-10-26

**Authors:** Hossein Kamran Haghighi, Fatemeh Sadat Hoseinian, Ana Maria Sastre

**Affiliations:** 1 Department of Mining Engineering, Amirkabir University of Technology, Tehran 1591634311, Iran; f_hoseinian@aut.ac.ir; 2 Department of Chemical Engineering, Universitat Politècnica de Catalunya, ETSEIB, Diagonal 647, 08028 Barcelona, Spain; ana.maria.sastre@upc.edu

**Keywords:** flotation, leach residue, lead, zinc, silver

## Abstract

Millions of tons of zinc plant leach residues (ZPLR) have been stockpiled in Iranian hydrometallurgical zinc plants during the last few decades. Due to the low grades of zinc, lead, and silver in these residues, these residues have been abandoned without treatment. The authors of this paper studied zinc plant leach residues (ZPLR) to propose a flotation process for separating and producing lead and silver concentrate. A response surface methodology (RSM) was employed to obtain six models for optimizing the best conditions for lead recovery, lead grade, zinc recovery, zinc grade, silver recovery, and silver grade. In these models, the effect of the different main variables, including density, flotation time, pH, sodium sulfide dosage, and potassium amyl xanthate dosage, was investigated to optimize grades and recoveries. The studied ZPLRs were categorized into two types based on the disposal time, including new and old residues. The chemical analysis showed that the grades of lead, zinc, and silver in the new residues are higher than in the old residues. In a previous mineralogical study, it was found that silver forms in lead and zinc minerals as a solid solution within their structures. The resulting 3D graphs showed that the interacting variables have significant effects on responses. The ANOVA analysis exhibited the order of model significance to be lead grade (F-value of 36.46) > silver grade (19.76) > lead recovery (7.88) > zinc grade (5.63) > silver recovery (5.58) > zinc recovery (4.83). Based on these models, under the conditions of 1126.26 g/cm^3^ density, 20.83 min retention time, 9.9 pH, 6 kg/t sodium sulfide, and 749.66 g/t potassium amyl xanthate dosage for a new residue type, the recoveries of lead, zinc, and silver were determined to be 51.10%, 11.13%, and 72.85%, with grades of 38.87% Pb, 8.46% Zn, and 1209.11 g/t Ag, respectively. According to the feasibility study results, the presented work is reasonable in terms of technical, economic, and investment potential.

## 1. Introduction

Zinc-lead residues are produced by zinc-lead extraction plants and are commonly treated using flotation and leaching processes to extract the zinc and lead. The zinc-lead industry has developed rapidly, due to the increasing demand for zinc-lead products [1,2,3]. Therefore, significant zinc-lead residues are dumped every year, which then leads to various critical environmental issues [4,5,6,7]. In addition, an increase in residue dumping leads to an enhanced volume of material being sent to landfills and facilities for residue storage and increases the costs of zinc-lead production [8,9,10]. These residues still contain valuable elements, including silver, lead, and zinc [11,12,13,14,15,16,17,18]. These old residues can be considered as a valuable resource for zinc and lead, which can now be extracted using new processes [9,19,20,21,22].

In recent decades, various studies have been carried out on recovering valuable elements from the residues of mineral processing plants. Most of the research on the recovery of lead and silver from the abovementioned residues is based on hydrometallurgy. Lorenzo-Tallafigo et al. [23] developed a hydrometallurgical process to recover zinc-copper by ferric leaching and lead-silver by hot brine leaching. The processes used in other studies [24,25,26,27], like the aforementioned study, are based on brine leaching. This method has several disadvantages, such as high water consumption, expensive chloride leach reagents, and ecological disruption [28]. In contrast to hydrometallurgical processes, some attempts have been made to recover metals from residues physically. Navidi Kashani and Rashchi (2008) evaluated the effects of chemical reagents on oxidized zinc flotation from a lead flotation residue. They obtained a 70% zinc recovery rate with a 40.7% grade under optimum chemical conditions [29]. Their results indicated that the depressant reagents have a significant effect on zinc flotation from residues. Yang et al. (2015) investigated zinc flotation from cyanide residues, using various activators to decrease the cyanide’s effect on the mineral depressant [30]. Their results showed that the zinc mineral can be successfully activated by certain activators, namely, sodium metabisulfite, hydrogen peroxide, and sodium hypochlorite. They indicated that the residues often contain strategic elements that can be reprocessed. Bagheri et al. (2020) studied sphalerite flotation from old residues with a high zinc grade [31]. They recovered 73% of the sphalerite by investigating different chemical conditions. However, a few papers have studied lead and zinc flotation from oxidized zinc ore leach residue. Among these papers, Rashchi, et al. [32] only worked on lead (anglesite) flotation from zinc leach residues by sodium sulfide at 7000 g/t, potassium amyl xanthate at 300 g/t, sodium silicate at 200 g/t, and pine oil at 60 g/t and a pH of 9.6. Lead grade and recovery were about 17% and 65% under these conditions, respectively. This work did not address the recovery of the silver contained in the leach residues. In addition, the study focused more on micro-flotation. Another paper studied the recovery of zinc and silver from zinc leach residue by flotation [33]. In the abovementioned study, zinc and silver were recovered after the water leaching of zinc leach residues by flotation at a pH of 3–5, 700 g/t C_4_H_9_O)_2_PSSNH_4_ (ammonium dibutyl dithiophosphate), 500 g/t (NaPO_3_)_6_ concentration, and a pulp density of 20% at 3 min. Based on these optimized conditions, a concentrate with 9256.41 g/t Ag and 12.26% Zn was obtained, from which the recoveries of silver and zinc were approximately 80.32% and 42.88%, respectively. Lei, Yan, Chen, and Xiao [6] worked on the flotation of zinc leach residues containing anglesite, quartz, and gypsum. They floated anglesite using salicylhydroxamic acid as a collector, an inhibitor of sodium silicate, and a frother of terpineol. The collector used in the abovementioned study is a particular collector for which accessibility is difficult. Other researchers chemically reduced the leach residues by coal or coke into products that could be floated by sodium diethyl dithiocarbamate or butyl xanthate [34,35]. The difficulty of the process, high energy consumption, and the high consumption of collectors are some disadvantages of these methods.

In Iranian zinc-lead processing plants, large amounts of residues have been produced, with noteworthy potential for reprocessing and the recovery of various elements such as lead and silver. Therefore, these residues can be considered secondary resources that can be recycled using advanced processes. According to the literature, there is no study on the simultaneous flotation of lead and silver from zinc leach residues. For the first time in the literature, the current study evaluates the flotation of lead and silver from two types of zinc-lead leach residues derived from zinc hydrometallurgical plants. In this regard, the effects of various main parameters on the process, such as density, flotation time, pH, sodium sulfide dosage, potassium amyl xanthate dosage (PAX), and residue types (both old and new) have been studied.

## 2. Materials and Method

First, the samples collected from the residues were divided into old and new types, divided according to the sampling date, and then they were sorted according to the numbering of the samples. During sampling, non-reliable samples were discarded. Then, the samples were categorized according to the lead grade into ranges below 1%, 1–2%, 2–3%, and 3–4%. Then, a representative sample was prepared from each batch. All samples were archived according to Table 1.

The flotation experiments were carried out on old and new representative residue samples obtained from a residue landfill site in Zanjan, Iran. The particle size distribution of each sample was analyzed using mechanically shaken Tyler sieves. X-ray diffraction (XRD) and atomic adsorption spectroscopy were used to determine sample mineralogy and perform chemical analysis, respectively. The archival sample with a lead grade below 1% was not examined due to the small quantity of lead it held and the impossibility of achieving the desired results via flotation experiments. Other samples were first subjected to quantitative analysis, the results of which are shown in Table 2.

The analysis obtained from the isolated samples, grouped according to the declared grades of the samples, shows that there is not much difference in the grades of the representative samples. This difference is a maximum of 1.14% for lead, 0.87% for zinc, and 43 ppm for silver. According to the results obtained from quantitative analysis of the samples, further investigations and flotation experiments were carried out on those samples in the range of 1–2%. The reason for this decision was, firstly, the abundance of these samples. Secondly, if satisfactory results were obtained from this range, the results could easily be generalized to higher grades. The experiments used sodium sulfide (Merck, Germany) as an activator, PAX (HJChem, China, CAS 2720-73-2 90%) as a collector, MIBC (Alfa Aesar, 99% C_6_H_14_O) as a frother, and lime (industrial grade, ZZIC Co, Iran) for pH adjustment. A Denver-type laboratory cell (Daneshfaravaran Co, Iran) was used for the flotation experiments, at a constant impeller speed of 1500 rpm.

### 2.1. Flotation Experiments

A pulp with the desired density was prepared and conditioned for 9 min. Then, sulfur sodium, PAX, and MIBC at the desired concentrations were added to the pulp and agitated for 3, 2, and 1 min, respectively. After each experiment ended, the concentrate and tail samples were filtered and dried to prepare them for weighting and chemical analysis.

### 2.2. Experimental Design

The effects of various main variables, including residue type (both old and new), pH, sulfur sodium dosage, PAX dosage, flotation time, and density, on the recoveries and grades of zinc, lead, and silver were studied using a response surface methodology. The variable levels presented in Table 3 were selected from the results obtained from the preliminary experiments.

To optimize the process, the independent variables’ effects upon the recoveries and grades of zinc, lead, and silver were modeled using the Design-Expert software (13.0.5.0 64 bit, 2021), based on the D-optimal response surface methodology (D-Optimal-RSM). The reasons for choosing this software were the consequent reduction of experimental runs, its being more feasible and efficient than other RSMs, more accurate correlation, and optimization with a maximizing information matrix (|X’X|) [36]. These independent and interacting variables were chosen according to model analysis (R^2^ and lack-of-fit analysis. Then, a one-way ANOVA analysis was used to evaluate the model’s significance (*p* < 0.05) [37,38,39]. The contour and surface response plots were evaluated to determine the various variables’ effects on the recoveries and grades of zinc, lead, and silver.

## 3. Results and Discussion

### 3.1. Characterization

**Figure 1:**
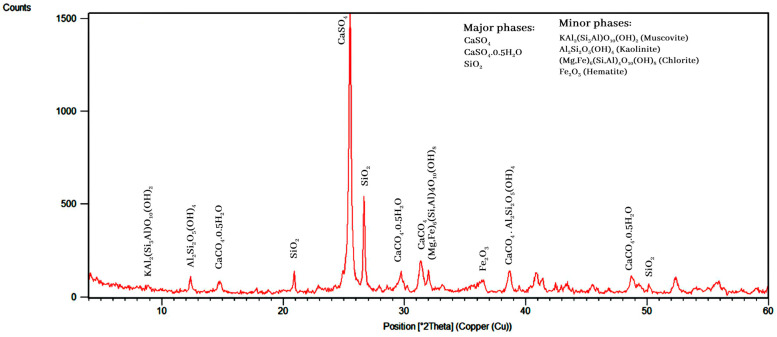
XRD analysis of the residues.

The XRD results indicated that quartz (SiO_2_), kaolinite (Al_2_Si_2_O_5_(OH)_4_), chlorite ((Mg,Fe)_6_(Si,Al)_4_O_10_(OH)_8_), hematite (Fe_2_O_3_), bassanite (CaSO_4_,0.5H_2_O), anhydrite (CaSO_4_), and muscovite-illite (KAl_2_Si_3_AlO_10_(OH)_2_) were the main minerals found in the residues (Figure 1). The mineralogical study indicated that these ores have no silver minerals, although silver can be present in lead and zinc mineral structures as a solid solution. Our earlier, similar study confirmed this finding [40,41]. The remarkable point in the XRD results is the effect of sulfuric acid, lime, temperature, and the reactions performed during the leaching process. The minerals in this sample included two categories of minerals that are both reactive and non-reactive with sulfuric acid. Anhydrite and bassanite minerals are formed as the result of acid and lime reactions. Quartz and hematite minerals do not react with acid and water. Other minerals, such as muscovite-illite, kaolinite, and chlorite, could derive from the residues of the silica ore. The absence of a peak for lead and zinc minerals is due to their low grades in the samples.

In order to determine the grades of the metals, atomic adsorption spectroscopy was used to analyze the digested solid residues in aqua regia. The results show that the grades of lead, zinc, and silver in the old and new residues were 1.06%, 1.50%, and 40 ppm, and 1.52%, 1.75%, and 66 ppm, respectively. The results indicated that the grades of lead, zinc, and silver in the new residue were higher than in the old residue. Old residues comprise those that were disposed of in the lower levels of the landfill, whereas the new residues were disposed of in the upper levels. The higher grade of the old residues can be attributed to the higher grade of the metals in the ore feeds used in the plants.

**Figure 2:**
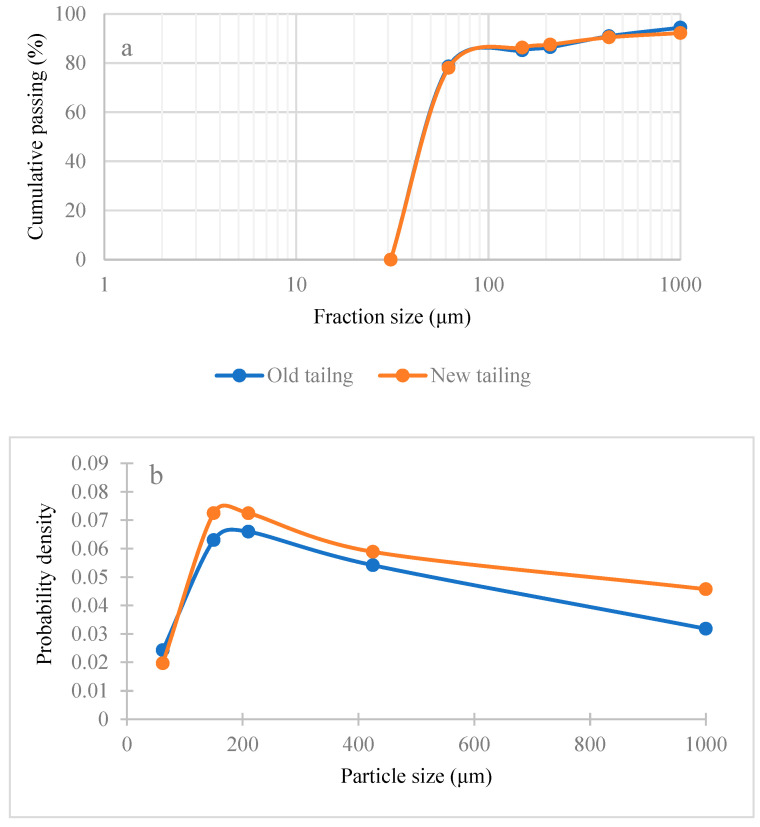
The particle size distribution of old and new samples in the form of (**a**) semi-logarithmic and (**b**) Gauss Bell curve.

**Figure 3:**
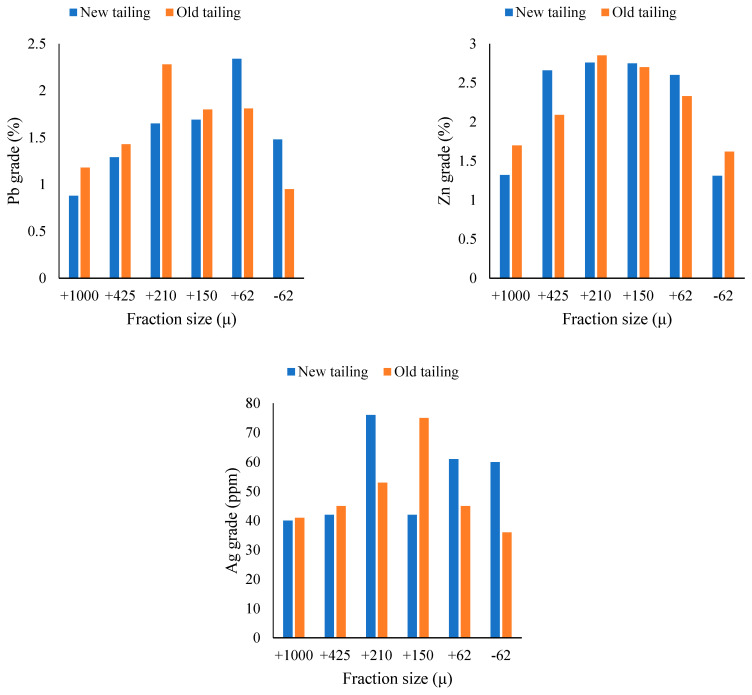
The grades of lead, zinc, and silver of the old and new residues according to fraction size.

The results of the particle size distribution of samples and Gauss bell curves are shown in Figure 2a,b. According to this Figure, the d_80_ values of both old and new residues are 62 microns. Thus, the samples were used in the flotation experiments without grinding. The chemical analyses of the fraction sizes of the old and new tailings are shown in Figure 3. The results show that the grades of lead, zinc, and silver are different in different fractions in the old and new tailings. The differences in the grades of lead, zinc, and silver in the different fractions may be due to differences in the performance of flotation and leaching processes in the plants. This could be due to the different conditions of chemical and hydrodynamic parameters in the research, such as pH, temperature, the presence of oxide and non-oxide minerals, fine particles obtained from the mill section, and the lack of liberation of the minerals.

### 3.2. Model Performance

Using the D-optimal design, 33 experiments were suggested by DX7 for six variables, namely, density (A), flotation time (B), pH (C), sodium sulfide dosage (D), PAX dosage (E), and residue type (F), as listed in Table 4; these were varied at the ranges presented in Table 3. The effects of these main variables on the recovery and grades of zinc, lead, and silver were evaluated by ANOVA analysis. A summary of the results obtained is shown in Table 5. The results indicate that all models were significant (the *p*-value of the models was less than 0.05, and their lack of fit was not significant (*p*-value > 0.05)). The adequate precision ratios of all models were higher than 4, which indicated an adequate signal for all models. Table 5 shows that all models for predicting the recovery rates and grades of lead, zinc, and silver were considered reasonable in terms of the R^2^, *p*-value, lack of fit, C.V.%, and adequate precision. The significant variables in each model have also been presented in Table 5. It should be noted that C.V.% calculates the closeness of the estimated values to the experimental (actual) values [42]. In contrast, R-squared, in the context of predictive accuracy, calculates the amounts of variability in the actual values estimated by the model [42].

The ANOVA analysis shown in this table presented the order of model significance as lead grade (F-value of 36.46) > silver grade (19.76) > lead recovery (7.88) > zinc grade (5.63) > silver recovery (5.58) > zinc recovery (4.83).

**Figure 4:**
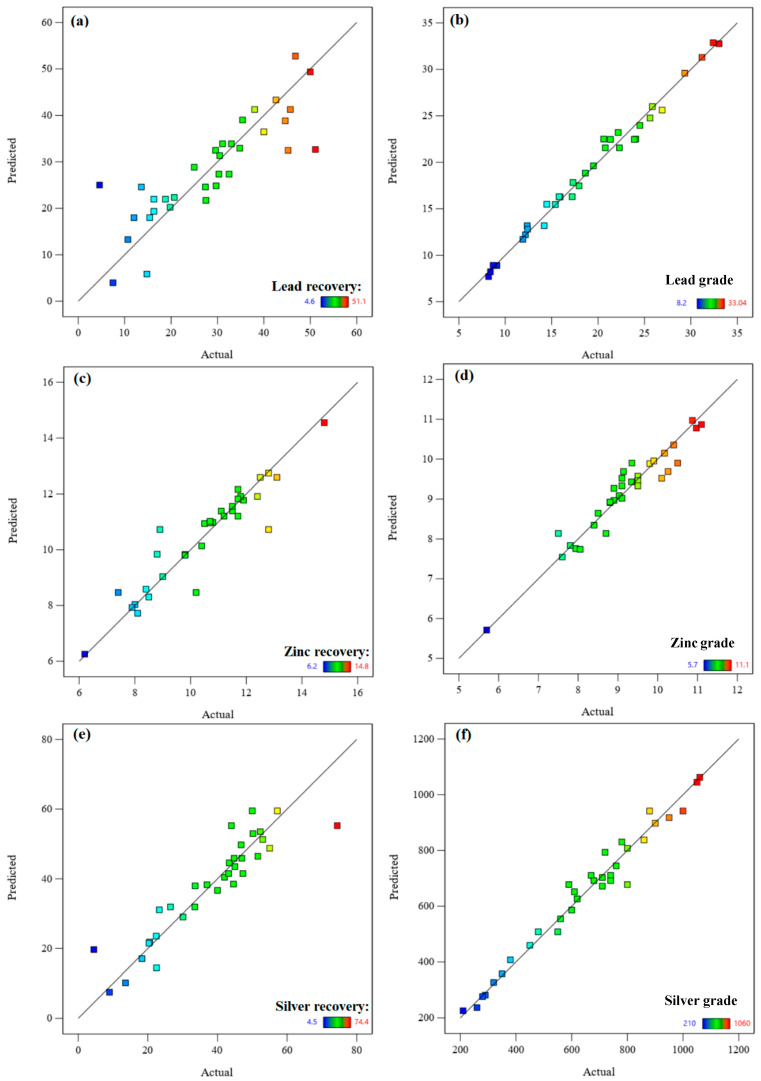
The actual values of recovery and grade of lead, zinc, and silver, as plotted here, versus the predicted values: (**a**) lead recovery, (**b**) lead grade, (**c**) zinc recovery, (**d**) zinc grade, (**e**) silver recovery, (**f**) silver grade.

The measured values of the recovery and grades of lead, zinc, and silver (actual values) versus their predicted values using the obtained models are plotted and shown in Figure 4. It is evident that the fitted regression equation obtained from the models indicates the appropriate fit of the models.

### 3.3. Effect of Parameters and Interactions

The six models developed by the D-optimal design of experiments (as shown in Table 4) represent the effects of the mentioned parameters (density (A), flotation time (B), pH (C), sodium sulfide dosage (D), PAX dosage (E), and residue type (F)) and their interactions on six responses (lead recovery, zinc recovery, silver recovery, lead grade, zinc grade, and silver grade). For this section, the effects were investigated in three binary categories of responses, namely, recovery-grade of lead, recovery-grade of zinc, and recovery-grade of silver. The interactions between independent parameters (density, flotation time, pH, sodium sulfide dosage, PAX dosage, and residue type) and responses (recovery and grades of lead, zinc, and silver) were investigated using 3D surface response plots.

#### 3.3.1. Recovery and Grade of Lead

The results show that density, flotation time, and residue type are not significant variables and have less effect on lead recovery, while pH, sodium sulfide dosage, PAX dosage, and the interactions of flotation time–sodium sulfide dosage and sodium sulfide dosage–residue type significantly affect the lead recovery rate (Table 5). The sodium sulfide dosage, PAX dosage, and interactions of density–flotation time, density–sodium sulfide dosage, density–PAX dosage, density–residue type, flotation time–pH, flotation time–sodium sulfide dosage, flotation time–PAX dosage, flotation time–residue type, sodium sulfide dosage–PAX dosage, and PAX dosage–residue type have a significant effect on the lead grade.

**Figure 5:**
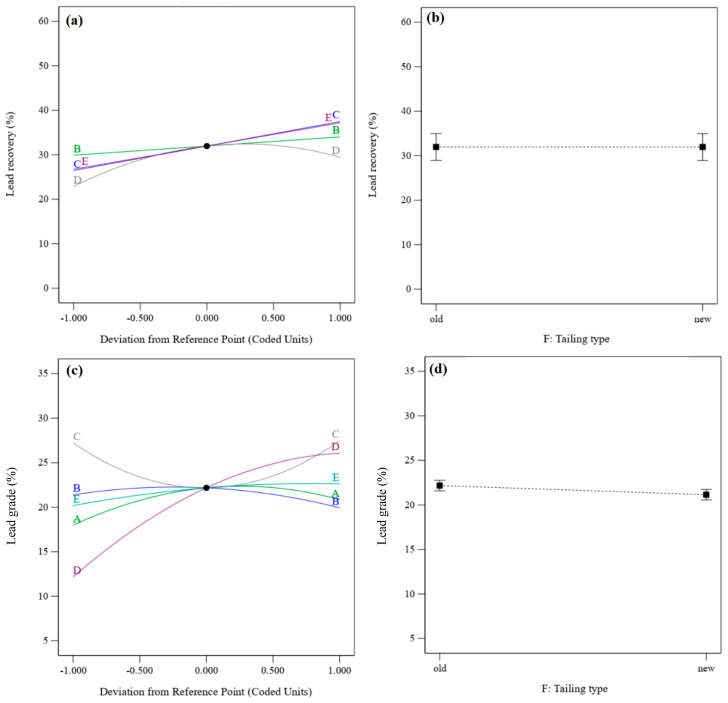
(**a**) Perturbation plot of lead recovery, (**b**) tailing type—effect on recovery, (**c**) perturbation plot of lead grades, and (**d**) tailing type—effect on lead grade.

Figure 5 shows the perturbation plots for lead recovery and lead grade. It presents the comparative effects of density, flotation time, pH, sodium sulfide dosage, and PAX dosage on lead recovery and lead grade under the conditions of density = 1200 g/cm^3^, flotation time = 17.5 min, pH = 9, sodium sulfide dosage = 4 kg/t, and PAX dosage = 500 g/t for the old residue type. Lead recoveries from both residues are approximately equal (Figure 5a,b), but the lead grade obtained from the old residue is slightly higher than from the new residue (Figure 5c,d).

**Figure 6:**
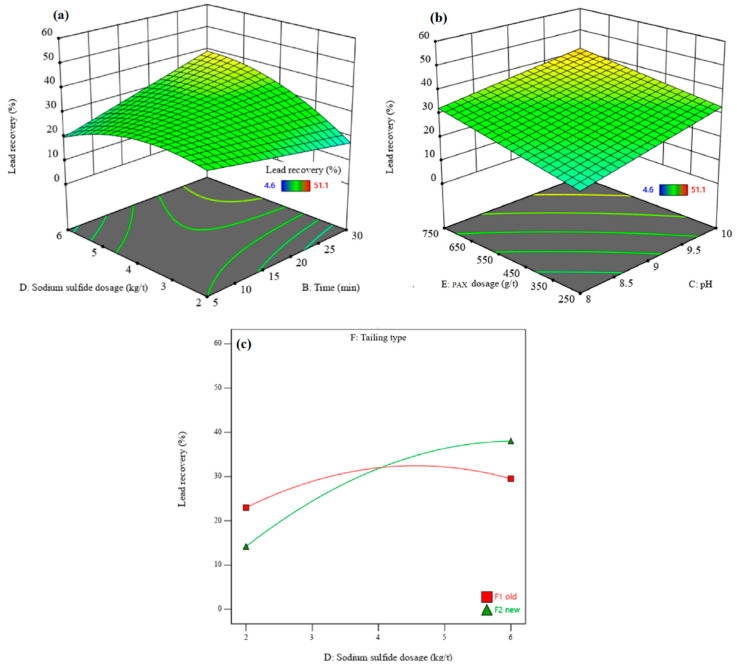
The interaction effects of sodium sulfide dosage–flotation time (**a**), PAX dosage–pH **(b**), and sodium sulfide dosage–residue type (**c**) on the lead recovery rate.

**Figure 7:**
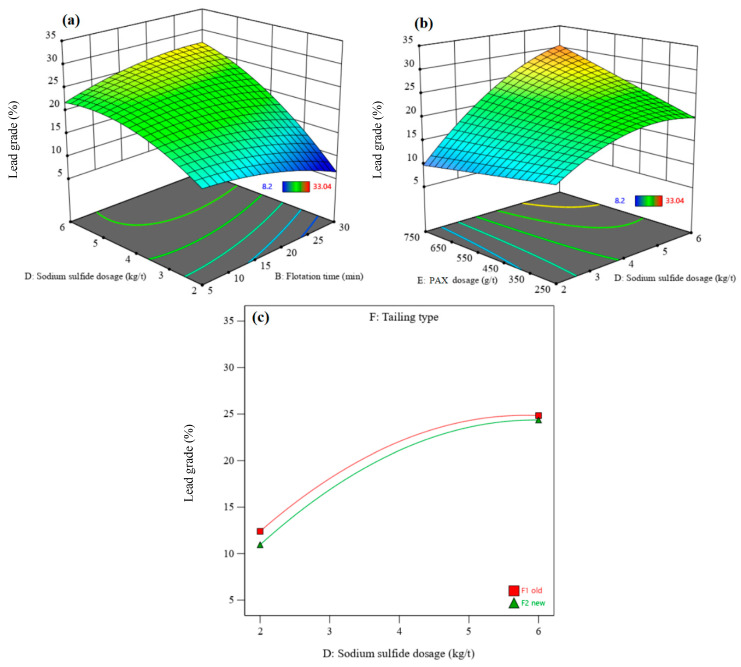
The interaction effects of sodium sulfide dosage–flotation time (**a**), PAX dosage–sodium sulfide dosage (**b**), and sodium sulfide dosage–residue type (**c**) on the lead grade.

The interactions between independent parameters (density, flotation time, pH, sodium sulfide dosage, PAX dosage, and residue type) and recovery and lead grade were investigated using 3D surface response plots. In this setup, two independent parameters change in the ranges designed, while the other parameters are constant. Figure 6 and Figure 7 show the 3D surface responses of lead recovery and lead grade obtained with the DX13 software, respectively. Figure 6a and Figure 7a indicate that the interaction effect of sodium sulfide dosage and flotation time on lead recovery and lead grade is significant. The lead recovery and grade are enhanced by increasing the sodium sulfide dosage and the flotation time (Figure 6a and Figure 7a). At a flotation time of 5 min, the lead recovery slightly increases with increasing sodium sulfide dosage, up to the optimum value, and then decreases while the lead grade increases. This is because the sulfide ions create a negative surface on the lead minerals, creating higher adsorption of xanthate molecules on mineral surfaces. Therefore, their floatability increases with increasing sodium sulfide dosage, up to the optimum value. Also, a higher sodium sulfide dosage can depress the sulfide minerals under some conditions and can lead to a decrease in the lead recovery rate. It is well known that at a higher sodium sulfide dosage, lead minerals are depressed since the sulfide ions are favorably absorbed by xanthate [43]. Furthermore, the excess sodium sulfide reduces the pulp’s potential and depresses minerals [44]. After a flotation time of 30 min, an increase in the sodium sulfide dosage from 2 to 6 kg/t leads to a significant increase in the lead recovery rate (approximately 20%) and lead grade (approximately 21%). This indicates that a longer flotation time is required to activate lead minerals. The lead sulfide minerals in the residues have been oxidized over time, leading to a decrease in their flotation kinetics [45]. With increasing flotation time, lead grade and recovery rates increase to the optimum value. After the optimum value, the lead grade decreases. With a lower retention time, only some easy-to-float minerals pass into the concentrate, causing an enhancement in grade and a reduction in recovery rate [43]. Lead recovery and grade increase with an increasing PAX dosage (Figure 6b and Figure 7b). In addition, sodium sulfide dosage and pH have a direct relationship with each other, due to sodium sulfide having alkaline properties. At a pH of 10, maximum lead recovery is obtained. Figure 6c shows the interaction effect of residue type and sodium sulfide dosage on lead recovery. The sodium sulfide dosage has a higher effect on lead recovery from new residue than from old residue. In comparison, sodium sulfide dosage has a slightly lower effect on the lead grade of new residue than on that of old residue (Figure 7c). Under the experimental optimum conditions, where density = 1104.91 g/cm^3^, flotation time = 26.93 min, pH = 9.98, sodium sulfide dosage = 5.76 kg/t, PAX dosage = 628.90 g/t, and with the new residue type, a maximum lead recovery rate of 52.48% with a lead grade of 35.96% can be achieved. Also, the high consumption of reagents is due to the solubility of lead from the lattice of anglesite [32,46], whereby the cationic lead dissolved in the pulp reacts with xanthate and forms PbX_2_, and this reaction continues until all lead cations are removed. Then, S^2−^ adsorbs on the anglesite surface to create PbS, followed by the adsorption of the remaining collector molecules onto it [46]. The lead dissolution in acidified residues is probably higher than that of natural minerals. In addition, the presence of excess sulfate ions (from the leachate of sulfuric acid), which dissolve easily in water, can disrupt the lead flotation process. The inverse effect of sulfate ions on galena flotation by xanthate was shown by Shahverdi et al. [47]. Their results highlighted that the increase in sulfate ions resulted in the formation of lead sulfate on the surface, which decreased the adsorption of xanthate. In this regard, sulfate acts as a barrier that restricts the contact between xanthate and the surface of the lead mineral.

#### 3.3.2. Recovery and Grade of Zinc

The ANOVA results (Table 5) show that the density, flotation time, pH, density–PAX dosage, flotation time–pH, sodium sulfide dosage–residue type, and PAX dosage–residue type have significant effects on zinc recovery. In addition, the flotation time, pH, sodium sulfide dosage, residue type, and interactions of density–sodium sulfide dosage, density–PAX dosage, density–residue type, flotation time–pH, flotation time–residue type, pH– sodium sulfide dosage, pH–residue type, and sodium sulfide dosage–residue type have significant effects on the zinc grade (Table 5).

**Figure 8:**
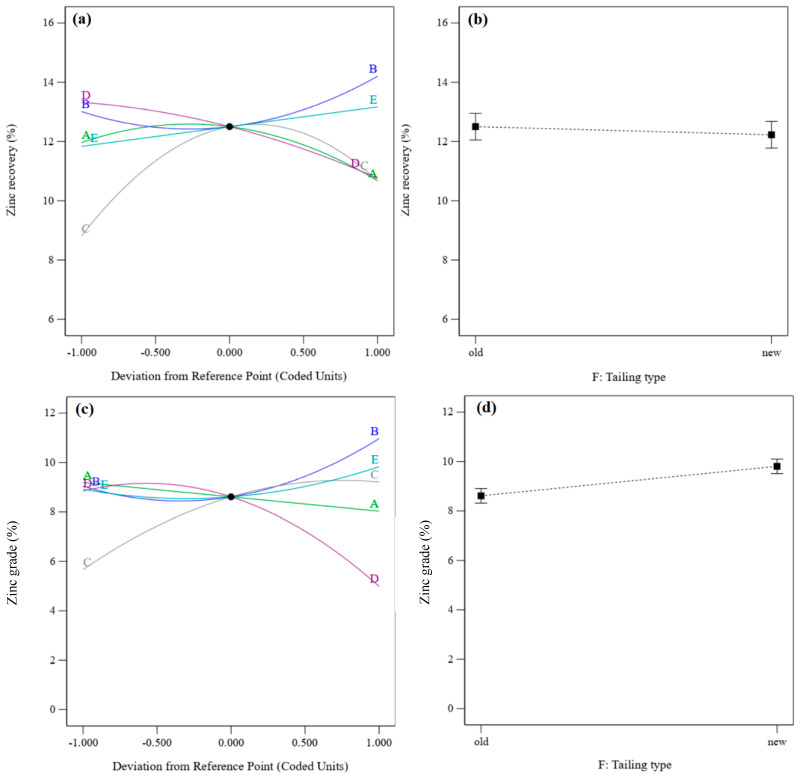
(**a**) Perturbation plot of zinc recovery, (**b**) the tailing effect on zinc recovery, (**c**) the perturbation plot for zinc grade, and (**d**) the tailing effect on zinc grade.

Figure 8 shows the perturbation plots of zinc recovery and zinc grade. It presents the comparative effects of effective parameters on zinc recovery and zinc grade under the conditions of density = 1200 g/cm^3^, flotation time = 17.5 min, pH = 9, sodium sulfide dosage = 4 kg/t, PAX dosage = 500 g/t, and the old residue type. Figure 8a shows that the zinc recovery rate and grade increase with increasing flotation time and PAX dosage. After increasing the pH, zinc recovery increases to the optimum value and then decreases. In contrast, the zinc grade increases with an enhancement of the pH value. With an increase in the sodium sulfide dosage, the zinc recovery rate decreases, and the zinc grade increases to the optimum grade and then decreases. Zinc recovery rates from the old residue are higher than those from the new residue (Figure 8b), but the zinc grade obtained from the old residue is lower than that from the new residue (Figure 5d).

**Figure 9:**
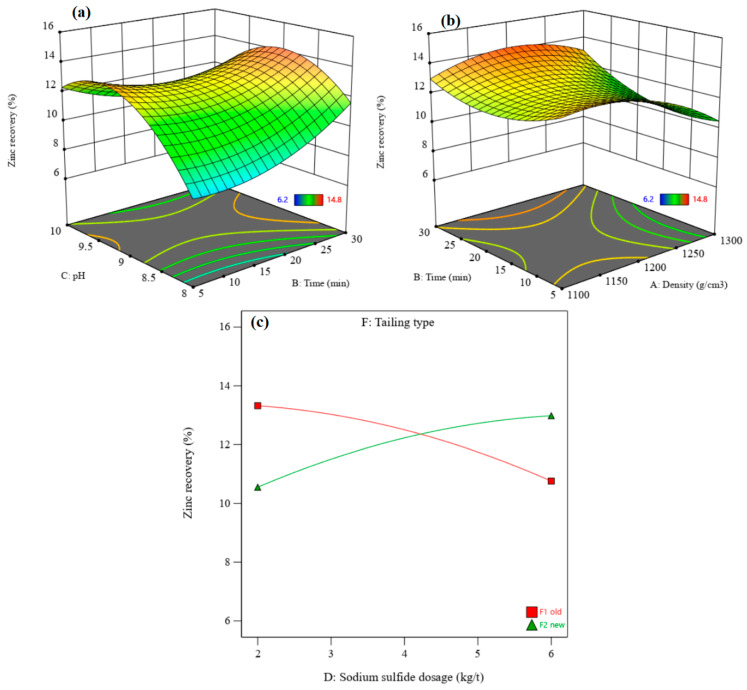
The interaction effect of pH–flotation time (**a**), flotation time–density (**b**), and sodium sulfide dosage–residue type (**c**) on the zinc recovery rate.

**Figure 10:**
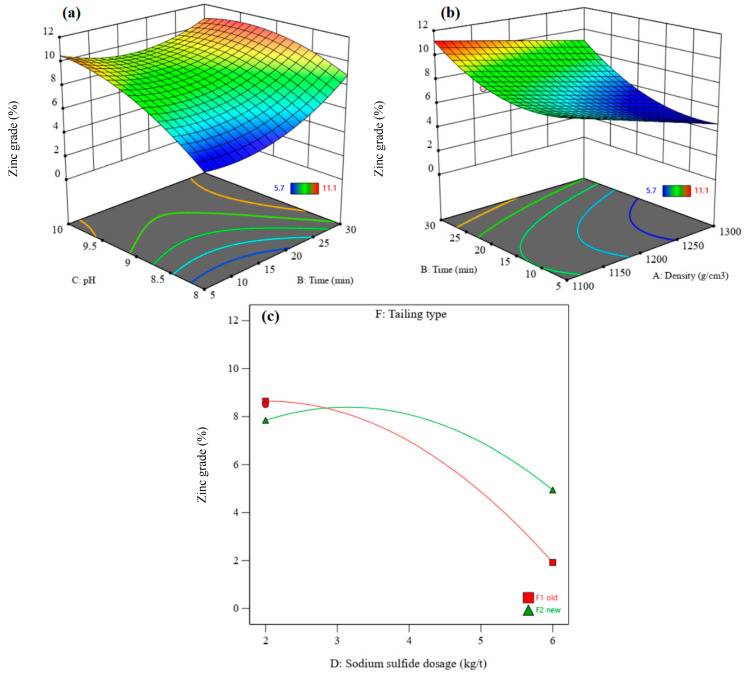
The interaction effect of pH–flotation time (**a**), flotation time–density (**b**), and sodium sulfide dosage–residue type (**c**) on the zinc grade.

Figure 9 and Figure 10 show the 3D surface response of zinc recovery and zinc grade, respectively. The interaction of pH and flotation time shows that zinc recovery increases after increasing the pH from 8 to 9–9.5, after which it decreases (Figure 9a). In contrast, the zinc grade increases with an enhanced pH (Figure 10a). Zinc sulfide minerals in the residues oxidize over time, leading to a decrease in their flotation kinetic. Thus, a higher flotation time is needed to float zinc minerals in order to achieve higher zinc recovery rates and zinc grades.

Figure 9b and Figure 10b show the interaction of flotation time and density on the zinc recovery rate and zinc grade, respectively. The zinc recovery rate increases with increasing density from 1100 to 1200 g/cm^3^; after that, it decreases with enhancing density to 1300 g/cm^3^. An increase in the density leads to a decrease in the zinc grade, due to increased gangue minerals and the associated entrainment. This suggests that zinc flotation performance may be improved by conducting the experiments at a lower density.

Figure 9c shows the interaction effect of residue type and sodium sulfide dosage on the zinc recovery rate. The sodium sulfide dosage has a negative effect on zinc recovery from old residue, while it has a positive effect on recovery from new residue. Increasing sodium sulfide dosage (in the new residue) increases the zinc grade to the optimum value and then decreases it (Figure 10c). The zinc grade decreases with increasing sodium sulfide dosage in the old residue. A decrease in the zinc flotation for old residues refers to the oxidation of these residues and the release of sulfide products such as sulfate or free sulfur, creating a depression condition with excess Na_2_S [43].

In the experimental optimum conditions, where density = 1204.92 g/cm^3^, flotation time = 28.88 min, pH = 8.96, sodium sulfide dosage = 4.51 kg/t, PAX dosage = 267.09 g/t, and the new residue type, a maximum zinc recovery rate of 16.12% with a zinc grade of 11.64% can be achieved.

#### 3.3.3. Recovery and Grade of Silver

The ANOVA results (Table 5) show that the density, pH, PAX dosage, and interactions of density–PAX dosage, flotation time–pH, pH–residue type, and sodium sulfide dosage–residue type have significant effects on the silver recovery rate. In addition, the density, flotation time, and sodium sulfide dosage and interactions of density–flotation time, density–pH, density–sodium sulfide dosage, density–PAX dosage, density–residue type, flotation time–pH, flotation time–sodium sulfide dosage, flotation time–residue type, pH–sodium sulfide dosage, pH–PAX dosage, sodium sulfide dosage–PAX dosage, and PAX–residue type have significant effects on the silver grade (Table 5).

**Figure 11:**
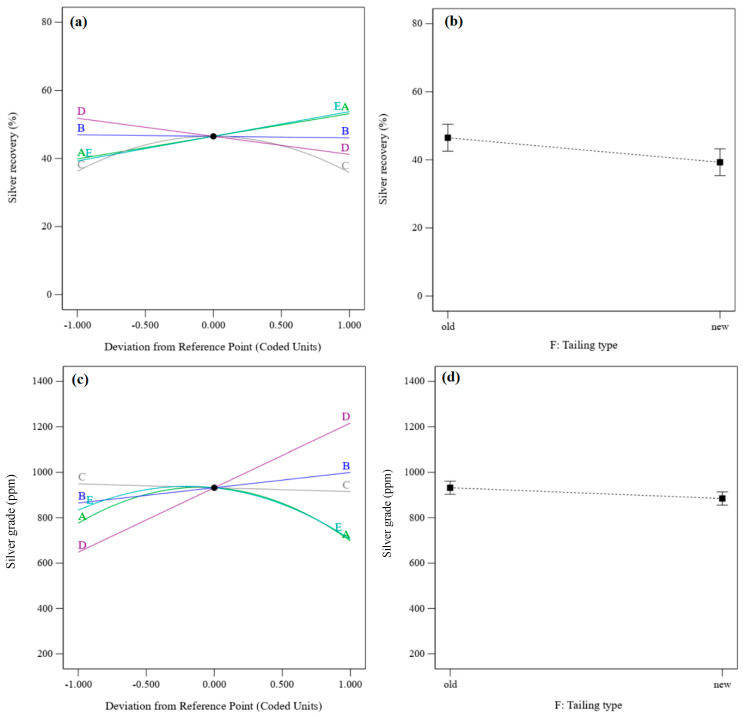
(**a**) Perturbation plot of silver recovery, (**b**) the tailing effect on silver recovery, (**c**) the perturbation plot of silver grade, and (**d**) the tailing effect on silver grade.

Figure 11 shows the perturbation plots for the silver recovery rate and silver grade. They show the comparative effects of parameters under the conditions of density = 1200 g/cm^3^, flotation time = 17.5 min, pH = 9, sodium sulfide dosage = 4 kg/t, PAX dosage = 500 g/t, and the old residue type. The results show that the silver recovery rate increases with increasing density and PAX dosage and decreases with increased flotation time and sodium sulfide dosage. It increases after increasing the pH to the optimum value and then decreases, while the silver grade decreases with increased flotation time and sodium sulfide dosage and increases after increasing the pH. It increases with an increase in the density and PAX dosage to the optimum value and then decreases. There is an inverse relationship between the effects of parameters on the silver recovery rate and silver grade. The silver recovery rate and grade in the old residue are higher than in the new residue.

**Figure 12:**
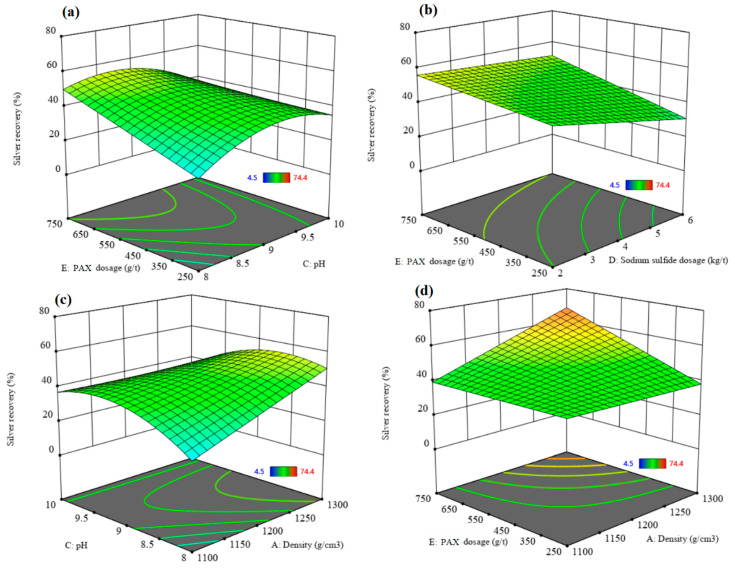
The interaction effect of (**a**) PAX dosage–pH, (**b**) PAX dosage–sodium sulfide dosage, (**c**) pH–density, and (**d**) PAX dosage–density on the silver recovery rate.

**Figure 13:**
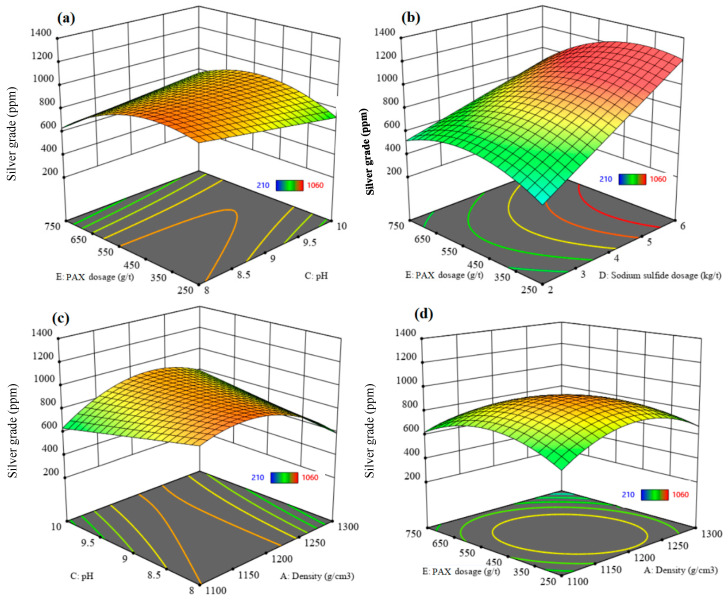
The interaction effect of (**a**) PAX dosage–pH, (**b**) PAX dosage–sodium sulfide dosage, (**c**) pH–density, and (**d**) PAX dosage–density on the silver grade.

Figure 12 and Figure 13 show the 3D surface response of silver recovery and silver grade, respectively. Figure 12a and Figure 13a indicate that excess PAX dosage increases silver recovery. In contrast, the silver grade increases to the optimum value and then decreases. An excess PAX dosage leads to the flotation of more gangue minerals into the concentrate, which decreases the silver grade. A slight decrease in the silver recovery rate is observed with increasing sodium sulfide dosage from 2 to 6 kg/t (Figure 12b), while a significant increase in the silver grade can be seen with increasing sodium sulfide dosage at a PAX dosage of 250 g/t (Figure 13b). It can be concluded that the higher silver grade obtained at a higher dosage of sodium sulfide might be due to the activation role of sulfide ions on the desired minerals.

Figure 12c and Figure 13c show the effect of the interaction of pH and density on the silver recovery and silver grade, respectively. The silver recovery increases after increasing the pH from 8 to 9.5; after that, it decreases very slightly when increasing the pH to 10. An increase in the density leads to an increase in the silver recovery rate, while the silver grade increases to the optimum value and then decreases due to the increased content of gangue minerals and the associated entrainment.

Figure 12d and Figure 13d indicate that increasing the PAX dosage increases the silver recovery rate while silver grade increases to the optimum value and then decreases. A higher silver recovery rate can be obtained at a higher PAX dosage and density. Conversely, higher silver grades can be obtained at the optimum values of PAX dosage and density. With a higher PAX dosage and density, more gangue minerals were floated into the concentrate, which decreased the silver grade. Under the experimental optimum conditions, where density = 1100 g/cm^3^, flotation time 20.81 min, pH = 9.66, sodium sulfide dosage = 6 kg/t, PAX dosage = 741.21 g/t, and the new residue type, a maximum silver recovery rate of 72.85% with a silver grade of 1285.23 ppm can be achieved.

**Figure 14:**
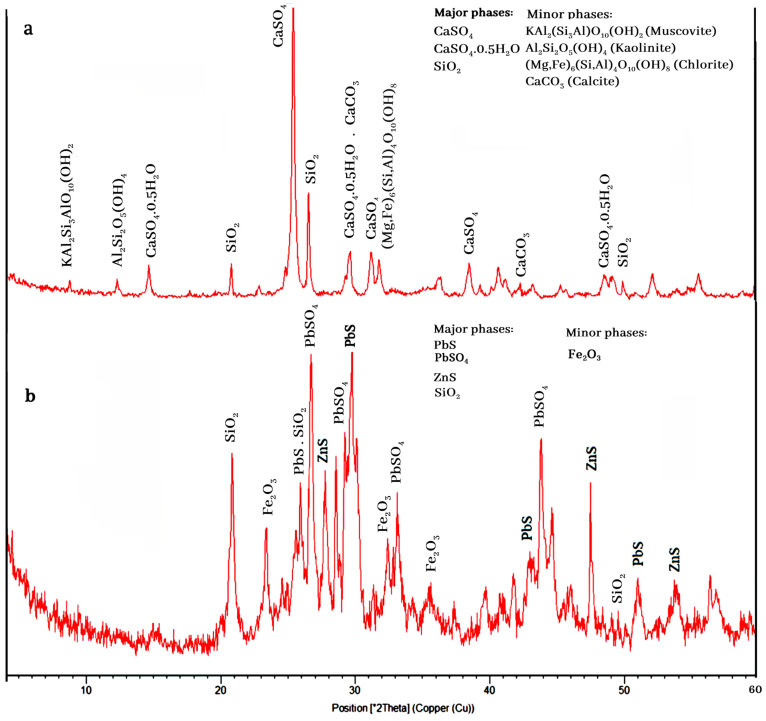
XRD analysis of flotation (**a**) tailing and (**b**) concentrate.

**Figure 15:**
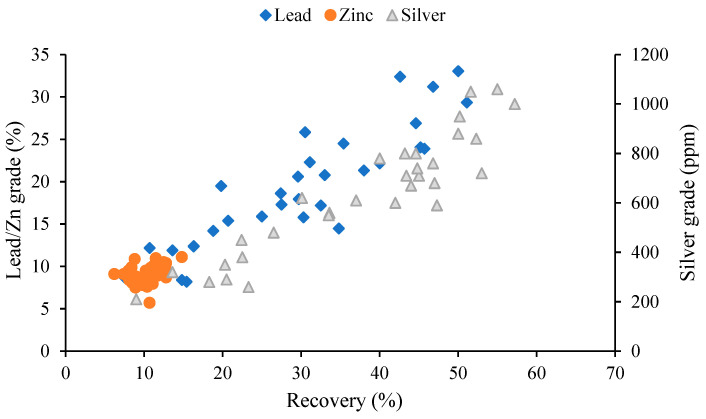
The recovery of lead, zinc, and silver, plotted versus their grades as obtained from the experiments.

The mineralogical study indicates that the ores have no silver minerals in them, but silver can be present in the lead and zinc mineral structures as a solid solution. Figure 14a,b shows samples of the general tailings and concentrates from one of the flotation tests conducted in an optimal state. As shown in Figure 14a, the minerals in the tailings are mainly calcium sulfate and quartz, with the minor phases of kaolinite, chlorite, muscovite, and calcite. The XRD analysis of the lead concentrate (Figure 14b) shows the main phases of anglesite, galena, sphalerite, and quartz. Figure 15 indicates that the trend of the lead recovery–grade figure is similar to that of the silver recovery–grade figure, resulting in the presence of silver ions in the sulfide and oxide lead mineral structures. Under the experimental optimum conditions of density = 1126.26 g/cm^3^, flotation time = 20.83 min, pH = 9.99, sodium sulfide dosage = 6 kg/t, PAX dosage = 749.66 g/t, and the new residue type, the recovery rates of lead, zinc, and silver achieved 51.10, 11.13, and 72.85%, with grades of 38.87, 8.46, and 1209.11 ppm, respectively.

### 3.4. Brief Economic Evaluation of a Lead and Silver Concentrate Production Plan by the Flotation Method

The preliminary assumptions for the estimation of cost, income, and profit are given in Table 6. It should be noted that the differences in product grades between Table 5 and the optimization results mentioned in the previous section are because the following results were obtained from mini-pilot experiments.

The price of concentrates in the marketplaces can be calculated, based on Equations (1) and (2):

The price of lead concentrate (USD/ton) = (Pb grade (%) − 3%) × LME of Pb–400$(1)

(2) $$\mathrm{The}\;\mathrm{price}\;\mathrm{of}\;\mathrm{silver}\;\mathrm{concentrate}\;(\mathrm{USD}/\mathrm{ton})=(\frac{\left(Ag(ppm)-50\right)}{31.10})\times (\mathrm{LME}\mathrm{of}\mathrm{Ag}–2\$)$$

The annual production of lead concentrate is 17,820 tons, and the total annual cost is USD 12,389,153. In this way, the total cost for each ton of lead concentrate will be USD 695.24. In contrast, the income obtained from each ton of concentrate is USD 875.

The income from the sale of the concentrate is USD 15,592,500. The annual production cost of lead concentrate is USD 12,389,153. As a result, the plan’s profit in the first year without tax will be USD 3,203,346, and, with 25% tax, it is USD 2,402,510. In addition, the total investment of the project is equal to USD 7,654,712 and the rate of return on profit with tax is 31%. Also, the rate of return on investment for a cake with an initial price of USD 2/kg will be 2.53 years. According to the obtained results, the presented plan is justifiable in terms of technical, economic, and investment potential.

## 4. Conclusions

Two types of old and new lead-zinc samples, prepared from the landfill of the Iranian zinc-lead plant residues, contained 1.06% lead, 1.51% zinc, 40 ppm silver, and 1.52% lead, 1.75% zinc, and 66 ppm silver, respectively. The effect of various parameters on the flotation recovery of lead, zinc, and silver from old and new zinc residues was studied. The Design-Expert software was employed to model the effects of the independent parameters upon the recoveries and grades of zinc, lead, and silver. To optimize the process, the effects of each parameter and their interactions on the recovery and grade of lead, zinc, and silver were investigated. The predictive accuracy of the models was evaluated according to the R-squared values, which were 72%, 99%, 88%, 92%, 85%, and 97% for lead recovery, lead grade, zinc recovery, zinc grade, silver recovery, and silver grade, respectively. At the experimental optimum conditions, which were density = 1126.26 g/cm^3^, flotation time = 20.83 min, pH = 9.99, sodium sulfide dosage = 6 kg/t, PAX dosage = 749.66 g/t, and new residue type, the recovery rates of lead, zinc, and silver were 51.10%, 11.13%, and 72.85%, with grades of 38.87%, 8.46%, and 1209.11 ppm, respectively. The mineralogical study indicates that the ores have no silver minerals. The results indicated that silver could be present in the lead minerals structures as a solid liquid because the trend of the lead recovery–grade figure is similar to that of the silver recovery–grade figure. The results indicated that the flotation process can recover lead and silver from the residues under optimum conditions. According to the feasibility study results, the flotation of zinc plant residues is justifiable in terms of technical, economic, and investment potential.

## Figures and Tables

**Table 1:** Classification of the samples taken from residue landfill.

Old	3 < Pb < 4	2 < Pb < 3	1 < Pb < 2	Pb < 1
New	3 < Pb < 4	2 < Pb < 3	1 < Pb < 2	Pb < 1

**Table 2:** Analysis results of the representative samples for each category.

Sample Category	Ag (ppm)	Zn (%)	Pb (%)	Moisture (%)
Old—Pb (3–4)	61	2.38	2.20	8.53
New—Pb (3–4)	51	1.20	1.71	11.95
New—Pb (2–3)	83	1.61	1.70	4.40
Old—Pb (2–3)	50	2.20	1.72	9.10
Old—Pb (1–2)	40	1.51	1.06	4.40
New—Pb (1–2)	66	1.75	1.51	4.80

**Table 3:** Variable levels used in the experiments.

Variables	Residue Type	PAX Dosage (g/t)	Na_2_S Dosage (kg/t)	pH	Flotation Time (min)	Density (kg/m^3^)
Levels	Old/new	250–700	2–6	Pb < 1	5–30	110–1300

**Table 4:** Experimental conditions, as designed by DX7 software.

Run	A: Density (g/cm^3^)	B: Time (min)	C: pH	D: Sodium Sulfide Dosage (kg/t)	E: Amyl Xanthate Dosage (g/t)	F: Ore Type
1	1100	20	8	2	577.50	old
2	1224	30	10	4.36	425	new
3	1100	30	10	2	750	new
4	1100	15	10	6	250	new
5	1100	30	9	3.96	250	old
6	1300	20	9	4.22	549.05	old
7	1152	30	10	6	727.50	old
8	1300	30	8	2	535	new
9	1100	5	9	4.52	725	old
10	1195	15	10	2	687.50	new
11	1206	10	8.5	2.26	741.64	new
12	1300	30	9	6	750	new
13	1198	5	9	6	454.61	new
14	1264	5	8.5	2.18	275	old
15	1300	5	10	2	362.50	new
16	1300	15	8	5	250	new
17	1290	5	10	6	250	old
18	1100	5	8	2.60	250	new
19	1181	25	9	2	250	new
20	1300	30	10	2	250	old
21	1218	30	8	3.6	750	old
22	1206	10	8.5	2.26	741.64	new
23	1210	10	10	4.94	750	new
24	1150	15	10	2.80	427.5	old
25	1224	30	10	4.36	425	new
26	1198	5	9	6	454.61	new
27	1100	20	9.5	4.36	577.5	new
28	1300	20	9	4.22	549.05	old
29	1295	5	10	2	750	old
30	1125.63	5	10	6	484.80	old
31	1210	15	9	6	750	old
32	1150	15	10	2.	427.50	old
33	1100	5	9	4.17	272.50	old

**Table 5:** The model performance for the recovery and grade of zinc, lead, and silver by statistical measures.

Model Type	F-Value of Model	*p*-Value of Model	Lack of Fit (*p*-Value)	C.V.%	R^2^	Adequate Precision	Significant Variables *
Lead recovery	7.88	<0.0001	0.1925	27.38	0.7243	11.8333	C, D, E, BD, DF
Lead grade	36.46	<0.0001	0.7676	7.31	0.9877	21.4947	D, E, AB, AD, AE. AF, BC, BD, BE, BF, DE, EF, A^2^, B^2^, C^2^, D^2^
Zinc recovery	4.83	0.0029	0.9919	10.13	0.8758	10.0156	A, B, C, AE, BC, DF, EF, A^2^, B^2^, C^2^
Zinc grade	5.63	0.0037	0.9549	6.05	0.9253	11.3558	B, C, D, F, AD, AE, AF, BC, BF, CD, CF, DF, B^2^, C^2^, D^2^, E^2^
Silver recovery	5.58	0.0007	0.7867	22.90	0.8481	8.3676	A, C, E, AE, BC, CF, DF, C^2^
Silver grade	19.76	<0.0001	0.3297	10.22	0.9705	15.8459	A, B, D, AB, AC, AD, AE, AF, BC, BD, BF, CD, CE, DE, EF, A^2^, E^2^

* A, B, C, D, E, and F are the density, flotation time, pH, sodium sulfide dosage, PAX dosage, and residue type, respectively.

**Table 6:** The initial assumptions of the economic evaluation.

1.8	Feed Lead Grade (%)	340	Number of Working Days per Year (Days)
1.55	Feed zinc grade (%)	3	Number of shifts per day
35	Feed silver grade (ppm)	8	Working hours per shift (hours)
40	Product lead grade (%)	2120	Daily input capacity (tons)
5	(%) Zinc grade in the product	17,820	Annual production of lead concentrate (tons)
800	Silver grade of the product (ppm)	52.40	Daily production (tons of concentrate)
1.48	The amount of zinc (%) left in the tailing	875	Price per ton of concentrate (USD)

## Data Availability

The original contributions presented in the study are included in the article, further inquiries can be directed to the corresponding author.
